# Masks of Albinism: Clinical Spectrum of Hermansky–Pudlak Syndrome

**DOI:** 10.3390/ijms252011260

**Published:** 2024-10-19

**Authors:** Anastasia M. Bobreshova, Sofya A. Ionova, Vitaly V. Kadyshev, Natella V. Sukhanova, Iuliia V. Viakhireva, Alexandra Yu. Filatova, Natalia V. Zhurkova, Peter A. Sparber, Andrey V. Marakhonov, Tatyana A. Vasilyeva, Olga A. Shchagina, Sergey I. Kutsev, Rena A. Zinchenko

**Affiliations:** 1 Research Centre for Medical Genetics, Moskvorechie Street, 1, Moscow 115522, Russia; lyusya.bobreshowa@gmail.com (A.M.B.);; 2 Petrovsky National Research Center of Surgery, Fotieva Street, 10, Moscow 119333, Russia

**Keywords:** Hermansky–Pudlak syndrome, albinism, ocular albinism, whole-exome sequencing, RNA analysis, ophthalmology

## Abstract

Hermansky–Pudlak syndrome (HPS) is a rare disease inherited in the autosomal recessive mode, including 11 clinical genetic subtypes. They are associated with impaired function of the BLOC protein complex (Biogenesis of Lysosome-related Organelles Complexes), and the subunits of the AP-3 complex (adaptor protein complex). Each has its own clinical features, but they are all characterized by albinism, bleeding disorder, and visual abnormalities. Eleven patients from eight unrelated families with an incoming diagnosis of albinism were examined and novel and previously described genetic variants in *HPS1*, *HPS6*, and *BLOC1S6* genes (types HPS1, HPS6, and HPS9) were found. To determine the optimal therapy and recommendations for further follow up, it is necessary to consider the entire clinical spectrum and genetic polymorphism of the disease. An interdisciplinary approach, combined with the use of non-routine diagnostic techniques such as RNA analysis, is essential for achieving accurate diagnoses in certain complex cases.

## 1. Introduction

Hermansky–Pudlak syndrome (HPS) (OMIM PS203300) is a rare autosomal recessive disease first described in 1953, characterized by a multisystemic presentation [1,2]. To date, 11 clinical genetic subtypes have been identified, each associated with pathogenic variants in the following genes: *HPS1*, *HPS3*, *HPS4*, *HPS5*, *HPS6*, *HPS11*, *AP3B1*, *DTNBP1*, *AP3D1*, *BLOC1S3*, and *BLOC1S6* [3,4]. These genes encode proteins which participate in the biogenesis of various organelles: lysosome, melanosomes and lysosome-related organelles (LROs). Consequently, HPS exhibits significant clinical heterogeneity [4,5].

The main symptoms shared by all HPS subtypes include increased bleeding tendency, ocular and oculocutaneous albinism, reduced visual acuity and nystagmus. Certain subtypes exhibit additional specific manifestations, such as bronchial system abnormalities (subtypes 1, 2 and 4), seizure episodes (subtypes 9 and 10), and granulomatous colitis (subtypes 2 and 6) [6]. Clinical features such as circulatory system involvement and secondary ocular changes may manifest at various ages during the first decade of life. Pulmonary fibrosis, a major cause of mortality of patients with HPS subtypes 1, 2 and 4, typically manifests in the third-to-fourth decade of life [3].

The highest prevalence of the HPS, attributed to a founder effect, has been reported in Puerto Rico, particularly in the northwestern part of the island, where the prevalence reaches 1:1800 individuals. This high prevalence is primarily due to mutations in the *HPS1* and *HPS3* genes [7,8]. Globally, the average frequency of HPS is estimated to range from 1:500,000 to 1:1,000,000 newborns [9].

The clinical presentation of HPS can overlap with other diseases, such as albinism and Chediak–Higashi syndrome (OMIM#214500) [10]. Genetic heterogeneity and clinical polymorphism complicate its diagnosis, making early confirmation crucial due to the presence of potentially life-threatening symptoms. In this article, we describe the clinical genetic characteristics of 11 patients from eight unrelated families initially presenting with suspected albinism. The diagnosis of HPS is based through genetic analysis.

## 2. Results

The cohort comprised 11 patients from eight unrelated families. Between 2016 and 2024, these patients were evaluated by a multidisciplinary team, including an ophthalmologist, dermatologist, geneticist, hematologist, and pulmonologist. All patients underwent molecular genetic testing, including high-throughput sequencing (HTS). The clinical data of these patients are summarized in Table 1.

The age of diagnosis in this cohort ranged from 4 months to 18 years (mean age 6.2 y). Upon examination, none of the patients exhibited dysmorphic facial features, mental or physical developmental abnormalities. However, all presented with characteristic features of the albino phenotype: white eyelashes, hair, and eyebrows, light blue eyes, and very light skin. An exception was noted in one patient, whose hair and skin were close to light brown.

Ophthalmological examination revealed horizontal nystagmus of varying severity and iris transillumination graded between 1 and 4 in all patients. Additionally, all patients demonstrated an absence of macular reflex and varying degrees of macular hypoplasia. Some patients exhibited atypical symptoms for HPS: Patient 1 presented with grade I microphthalmia, while Patients 2.1 and 2.2 displayed focal pigmentation at the periphery of the fundus.

Hematological abnormalities were observed in more than half of our patients (7/11), including prolonged bleeding time (presenting as extended epistaxis and bleeding following tooth extractions), bruising with minor injuries, and the presence of blood clots in the feces. Only two patients exhibited early signs of bronchopulmonary involvement. Notably, clinical polymorphism was observed within families, even among siblings carrying the same genetic variant. For example, Patient 2.2 experienced a bleeding disorder and an episode of obstructive bronchitis, in contrast to Patient 2.1. Similarly, Patient 6.1 and 6.2 displayed differences in the type of nystagmus and bleeding history.

Molecular genetic diagnosis for most patients started with a next-generation sequencing (NGS) panel targeting genes associated with albinism—*TYR*, *OCA2*, *MC1R*, *MITF*, *SLC45A2*, *TYRP1*, and *GPR143*—as the initial clinical diagnosis for all patients was albinism. However, no pathogenic variants were identified in these albinism-associated genes; therefore, albinism was not confirmed for all patients.

Subsequently, all patients underwent whole-exome sequencing (WES), which revealed variants in HPS-associated genes. The previously described variants were detected: NM_000195.5(*HPS1*):c.1189delC, NM_000195.5(*HPS1*):c.397G>T, and NM_000195.5(*HPS1*):c.972dupC. Additionally, novel variants were discovered, including NM_024747.5(*HPS6*):c.1133T>G, NM_000195.5(HPS1):c.938-1G>A, NM_024747.5(*HPS6*):c.241G>T, NM_024747.5(*HPS6*):c.1457C>T, and NM_012388.3(*BLOC1S6*):c.224+1G>A.

In Patient 4, WES revealed a single, previously described variant, NM_000195.5(*HPS1*):c.1189delC, with no additional potential causative variants identified. Since the second variant might be located deep within the *HPS1* introns and result in an aberrant mRNA structure, we developed an RNA analysis system assay targeting exons 2-20 of the *HPS1* mRNA to identify it. Total RNA was isolated from peripheral blood mononuclear cells (PBMCs) obtained from proband 4 and his parents. The RNAs were reverse transcribed, and the targeted locus was amplified and sequenced using the NGS platform. Deep NGS sequencing of the PCR product allows for us to make a comprehensive analysis of splicing alteration, allelic disbalance, and segregation data.

**Figure 1:**
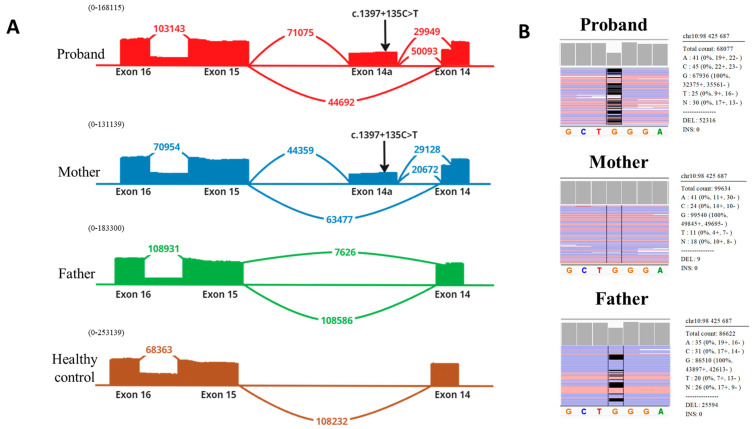
RNA analysis of HPS1 mRNA in family 4. (**A**) Deep NGS sequencing revealed the inclusion of additional exon 14a with c.1397+135C>T variant, which results in the formation of preterm stop codon. Allelic disbalance can be observed in the mother’s sample (wt:mut ratio 72:28), indicating degradation of transcripts with exon 14a via the NMD mechanism. (**B**) Allelic disbalance caused by the variant c.1189delC. In the father’s sample, wt:mut ratio is 77:23, indicating the degradation of transcripts via the NMD mechanism.

Analysis of deep mRNA sequencing data in the proband confirmed the c.1189delC variant, previously identified by whole-exome sequencing. This variant was detected at the RNA level in a heterozygous state (wt:mut ratio 56:44). The same variant was found in the proband’s father, with a wt:mut ratio of 77:23, indicating that transcripts with this variant undergo nonsense-mediated decay (NMD) (Figure 1).

Further analysis of the mRNA structure in the proband revealed the inclusion of an additional exon, 14a, between exons 14 and 15. Pseudoexon 14a contained the NM_000195.5(*HPS1*):c.1397+135C>T variant in a homozygous state (Figure 1A). This variant had not been previously described in patients with HPS and has a frequency of 0.003186% in gnomAD. Interestingly, the variant is located outside of canonical splicing site regions and is not predicted by bioinformatics splicing prediction programs (including SpliceAI). However, the ESE Finder program predicts a (SRSF6) binding site for splicing the regulator protein in this region. It was also noted that the inclusion of exon 14a led to the usage of two donor splicing sites of intron 14—the wild-type site and an alternative cryptic weak site.

The inclusion of pseudoexon 14a results in a frameshift and the creation of a premature stop codon (p.(Glu466Aspfs27) or p.(Ser459Thrfs1) when using the alternative donor site). The inclusion of pseudoexon 14a was also observed in the mother’s samples, with a wt:mut ratio of 72:28, further supporting the conclusion that transcripts containing pseudoexon 14a are degraded via NMD (nonsense-mediated decay).

To confirm that the inclusion of pseudoexon 14a is associated with the identified regulatory variant, we performed additional Sanger sequencing of the genomic locus containing the pseudoexon in the proband. No additional variants were detected except for NM_000195.5(*HPS1*):c.1397+135C>T.

**Figure 2:**
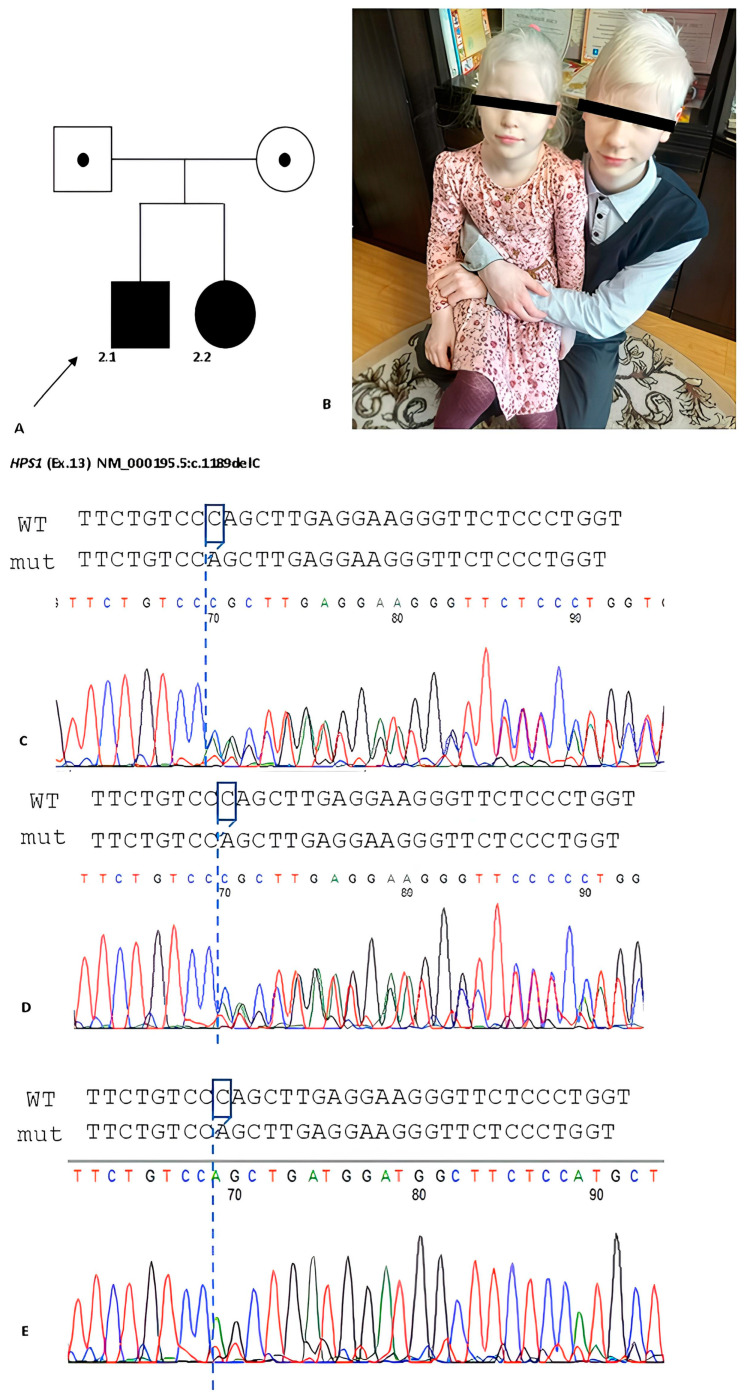
(**A**) The pedigree with two patients with HPS type 1. (**B**) Patients 2.1 and 2.2 have albinotic phenotype: white hair, eyebrows, white skin. (**C**–**E**) Electrophoregrams demonstrate heterozygous state of probland’s parents and homozygous variant of the probands.

**Figure 3:**
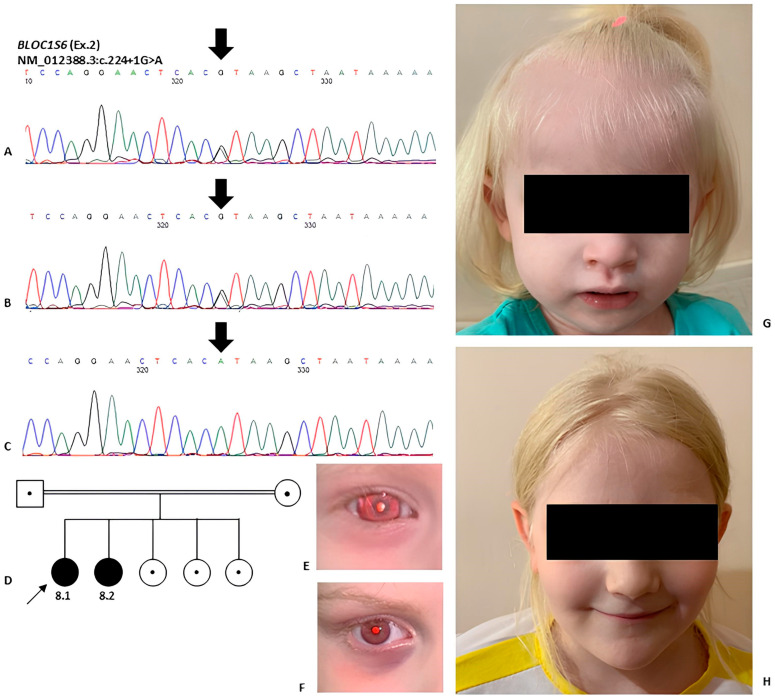
(**A**,**B**) Electrophoregrams demonstrate heterozygous state of variant NM_012388.3:c.224+1G>A in parents of probands. (**C**) Homozygous variant in proband and siblings in gene *BLOC1S6* with HPS type 9. (**D**) Pedigree from two generations of the probands 8.1 and 8.2 family with HPS type 9. (**E**–**H**) Photos of Patients 8.1 and 8.2 shows albinotic phenotype: blue eyes, white hair, eyebrows and skin.

The summary results of molecular genetic testing are presented in Table 2. Further segregation analysis was performed for families where parental samples were available. This analysis confirmed the biallelic state of the identified variants (Figure 2 and Figure 3). Utilizing HTS methods, six patients from five unrelated families were diagnosed with HPS type 1, three patients from two unrelated families with HPS type 6, and one family with two siblings with HPS type 9.

## 3. Discussion

Hermansky–Pudlak syndrome is a rare disease, with an estimated prevalence ranging from 1:500,000 to 1:1,000,000 newborns. It is characterized by considerable clinical polymorphism and genetic heterogeneity. Albinism, which includes hypopigmentation of the hair and skin, and ocular abnormalities, is often the initial diagnosis made shortly after birth. However, other HPS symptoms typically manifest later, which complicates and delays the diagnosis of HPS. Coagulation abnormalities may become apparent when patients begin walking or during the transition of teeth [13]. Beyond bruising, coagulation disorders can also present with intestinal and nasal bleeding, heavy menstruation, gum bleeding, and significant blood loss following surgical procedures [14,15,16,17]. Electron microscopy is the primary diagnostic tool for identifying blood system abnormalities, since changes in coagulation profiles and general blood tests are not always specific [18].

None of our patients exhibited dysmorphic facial features, birth injuries, or signs of impaired cognitive or physical development, consistent with the literature [18,19]. Ocular anomalies observed included nystagmus of various types and degrees of severity, iris transillumination, absence of macular reflex, and macular hypoplasia. These symptoms are commonly associated with various types of HPS and do not correlate specifically with the type of mutation or affected gene [20].

Additionally, our patients exhibited previously undescribed clinical manifestations including grade I microphthalmia (Patient 1), preservation of macular differentiation (Patient 3), and focal pigmentation retention in patients from the same family (Patients 2.1 and 2.2). They also presented non-classical symptoms of HPS, such as renal failure, schizophrenia, asthma, dextrocardia, and leukopenia, highlighting the clinical heterogeneity of HPS, which are described in the literature [21,22]. The clinical and genetic findings illustrate that despite the underlying genetic etiology, HPS presents with highly individualized manifestations, reflecting a broad spectrum of clinical heterogeneity both within the same family and between unrelated individuals, as corroborated by other studies [23,24].

Bronchopulmonary changes, which typically manifest between the third and fourth decades of life and progress to pulmonary hypertension, were not seen in our patients. At the initial stage of genetic counseling, it is crucial to establish strategies for ongoing monitoring and treatment. The literature data suggest that pirfenidone may improve progressive pulmonary fibrosis symptoms [25,26]. There are also literature data supporting the use of nintedanib followed by lung transplantation [27]. However, a consensus on the treatment of pulmonary fibrosis in HPS patients is lacking, and there is no established correlation between mutation type, affected gene, and of the onset of lung fibrosis [28]. General recommendations include symptomatic treatment and preventive measures: wearing sunglasses and sun-protected clothing, applying sunscreen on exposed areas of the skin, and regular examination by specialists such as pulmonologists, dermatologists, ophthalmologists, hematologists, therapists, and gastroenterologists.

Patients were admitted with a primary clinical diagnosis of “Albinism” and the main clinical symptoms were hair and skin hypopigmentation and eye pathology. So, the first step of molecular genetic diagnosis was the NGS panel sequencing of albinism-associated genes. After that, we performed WES because we assumed the presence of rare syndromic forms, which helped us to confirm the diagnosis. HPS is characterized by clinical and genetic heterogeneity and overlapping with other disorders, especially with isolated forms of albinism, so the HTS methods, such as WES, are more recommended for diagnosis [29,30].

In the cohort studied, the majority of patients (6 out of 11) were diagnosed with HPS type 1, 3 patients with HPS type 6 (3 out of 11), and 2 patients with HPS type 9 (2 out of 11). Retrospective studies of other populations indicate that HPS type 1 is the most prevalent [31,32]. Some populations are characterized by the accumulation of certain genetic variants. For instance, in Puerto Rico, 16 bp deletion in the *HPS1* gene and 3904 bp deletion in the *HPS3* gene are most common. Analysis of the Chinese population reveals that HPS type 1 is also the most frequent, affecting 47.5% patients, followed by HPS type 6, which affects 20% of patients [33].

The pathogenesis of HPS is primarily attributed to defects in the subunits of the AP-3 complex and biogenesis of the lysosome-related organelle (BLOC) complex [34,35,36]. These complexes are crucial for the transport of endosomes (melanosomes, dense granules, Weibel–Palade bodies, and large dense granules) both to the extracellular space and between intracellular compartments [37,38,39]. The differential involvement of these protein complexes in endosomal transport and biogenesis accounts for the varied clinical manifestations of HPS, both among different subtypes and between individual patients [12,40].

Various predictive software tools were used to assess the pathogenicity of the variants identified in our study. Previously described single-nucleotide deletion in exon 13 (NM_000195.5:c.1189delC) of the *HPS1* gene, a nonsense variant in exon 5 (NM_000195.5:c.397G>T) and small duplication in exon 11 (NM_000195.5:c.972dupC), all result in a shift in the open reading frame and formation of the premature stop codon [12]. Variant c.1189delC is cataloged in the ClinVar database (ID:21091) as a pathogenic variant and is found in the gnomAD database (v2.1.1) with a frequency of 0.006753%, and in the RuSeq database with a frequency of 0.09% [41,42,43]. The relatively high frequency of variant c.1189delC (7 from 10 alleles) in a studied cohort suggests a potentially high frequency of this variant among the Russian population, though further research is required. Frameshift variant c.972dupC is described in the Clinvar database (ID:5278) and is present in the gnomAD database with a frequency of 0.02620% and in the RuSeq database with a frequency of 0.136% [41,42,43]. Nonsense variant c.397G>T is not listed in gnomAD and RuSeq databases but is registered in ClinVar (ID 5281) as a pathogenic variant. These variants are predicted to cause nonsense mediated decay (NMD), as confirmed by the NMDEscPredictor program, and functional analysis by Shotelersuk et al. indicates a lack of RNA product from this allele [12,44]. Novel variant (NM_000195.5:c. c.938-1G>A) in the *HPS1* gene has not been found in gnomAD, ClinVar and RuSeq databases [41,42,43]. Bioinformatics algorithms (spliceAI, mmsplice, squirls, and spip) predict that this variant disrupts the canonical donor splice site, affecting the normal splicing pattern. According to ACMG guidelines, the c.938-1G>A variant should be classified as pathogenic (PM2, PVS1, and PP4).

Initially, molecular diagnosis was not established in family 4. However, the clinical picture and the presence of one pathogenic variant in the *HSP1* gene led us to propose the existence of a second variant that might be located outside the gene’s exons. We decided to perform RNA analysis, which has proven to be effective in such cases [45,46]. Indeed, through RNA analysis, we identified the intronic variant c.1397+135C>T, which led to the activation of a pseudoexon and a frameshift. This pathogenic mechanism is rare, and such an event has been described by us for the first time for this disease.

The proteins encoded by the *HPS1* and *HPS4* genes are essential subunits of the BLOC-3 complex, a key regulator of vesicle transport. This predominantly cytosolic complex is involved in transporting proteins necessary for the maturation of melanosomes, dense granules, and their subsequent exocytosis [47]. The loss of one of the subunits destabilizes the protein complex, leading to impaired melanosome maturation, which results in ocular albinism [36].

The novel variants in the *HPS6* gene identified in our patients are missense variants. Specifically, c.1133T>G and c.241G>T have not been found in the gnomAD database (v2.1.1) but are present in the RuSeq database with frequencies of 0.05931% and 0.02111%, respectively [43,47]. The missense variant c.1457C>T has been found in gnomAD and RuSeq databases with frequencies of 0.005968% and 0.04168%, respectively [43,47]. Predictive tools such as MutationTaster, MetaRNN and PolyPhen-2 suggest that the c.1133T>G variant affects the protein’s structure or function, while the pathogenicity of c.241G>T and c.1457C>T variants are ambiguous across different predictors. These variants are associated with the family’s phenotypes and involve amino acid substitution at highly conservative positions. According to ACMG guidelines, c.241G>T and c.1457C>T variants should be classified as variants of uncertain clinical significance (VUS) based on criteria PM2 and PP4, while the c.1133T>G variant should be considered as probably pathogenic (PM2, PP3, PM1, PP4). Patients with HPS type 6 exhibit a milder phenotype. The *HPS6* gene product is associated with the BLOC-2 protein complex involved in the transporting protein, including TYRP1 from the cell periphery to endosomes for further melanosome maturation [48,49]. Additionally, this protein complex contributes to the formation of Weibel–Palade bodies, which contain the von Willebrand factor, explaining the bleeding tendency in these patients [50].

Novel variant c.224+1G>A in the *BLOC1S6* gene has not been found in gnomAD and RuSeq databases [43,51]. Bioinformatics algorithms (spliceAI, mmsplice, squirls, and spip) predict that this variant disrupts the canonical donor splice site, affecting the normal splicing pattern. This variant segregates with the phenotype in a consanguineous family with two affected children and normal parents. According to ACMG guidelines, the c.224+1G>A variant should be classified as pathogenic (PM2, PVS1, PP1M, PP4). Experimental data from mouse models support this classification, demonstrating that the absence of a component of the BLOC-1 complex leads to destabilization of the entire complex [52,53,54]. The primary function of BLOC-1 is to facilitate the formation of the tubular structures necessary for the endosomal transport within the endosomal compartments [55,56]. In addition to changes in tubular structures, BLOC-1 deficiency in mice has been associated with changes in neurotransmitter levels in hippocampal cells, as well as alterations in behavior and cognitive functions [57].

## 4. Materials and Methods

### 4.1. Patients

The cohort consists of 11 patients from 8 unrelated families, all initially diagnosed with albinism at the Research Centre for Medical Genetics. The patients were examined by ophthalmologists and geneticists. Other specialists conducted examinations as needed based on the patients’ place of residence.

### 4.2. Informed Consent

Informed consent was obtained, as appropriate, from 11 subjects and/or their legal representatives included in the study. The study was conducted in accordance with the Declaration of Helsinki and approved by the Institutional Review Board of the Research Centre for Medical Genetics, Moskvorechie str., 1, Moscow, Russia.

### 4.3. DNA Extraction

Genomic DNA was isolated from peripheral blood cells using the Magen^®^ Genomic DNA Extraction Kit (Magen Biotechnology Co., Ltd., Guangzhou, Guangdong, China), according to the manufacturer’s recommendations.

### 4.4. Gene Panel Sequencing

The DNA analysis of the proband was performed on Ion S5 NGS sequencer (ThermoFisher Scientific, Waltham, MA, USA)with an average coverage of at least 240×. For the preparation of samples, an ultra-multiplex PCR technology AmpliSeq (Waltham, MA, USA) was used, coupled with subsequent sequencing. The analysis was carried out using a custom gene panel. The processing of the sequencing data is carried out using a standard automated algorithm Torrent Suite (ThermoFisher Scientific, Waltham, MA, USA), and by the GeneTalk software (https://www.gene-talk.de/) (GeneTalk GmbH, Bonn, Germany). To estimate the population frequencies of the identified variants, a cohort of projects, “1000 genomes”, ESP6500 (accessed on 1 August 2024), and GnomAD (v.2.1.1), were used. To assess the clinical relevance of the identified variants, the OMIM database (accessed on 13 March 2024), pathogenic variant database HGMD Professional version 2022.1, specialized databases on individual diseases and literature data were used.

### 4.5. Whole-Exome Sequencing

The DNA analysis of the probands was carried out on the NGS BGISEQ-500 (MGI, Shenzhen, China) using the method of pair-end reading (2 × 100 b.p.) with an average coverage of at least 70–100×. The selective capture of MGIEasy Exome Capture V4 (MGI, Shenzhen, China) DNA sites related to coding regions of more than 20,000 genes (59 MB) was used for sampling. Processing of sequencing data was carried out using an automated algorithm (the algorithm includes evaluation of sequencing quality, alignment of reads on the reference sequence of the human genome (GRCh37/hg19), postprocessing of alignment, identification of variants and filtering of variants by quality, annotation of the revealed variants on all known transcripts of each gene from the RefSeq database (accessed on 13 March 2024) using a number of methods to predict pathogenicity of substitutions (SIFT, PolyPhen2-HDIV, PolyPhen2-HVAR, MutationTaster, LRT, BMut), as well as methods of calculation of evolutionary conservation of positions (PhyloP, PhastCons). To estimate the population frequencies of the identified variants, samples of projects “1000 genomes”, ESP6500 and GnomAD v.2.1.1 were used.

### 4.6. PCR and Sanger Sequencing

Polymerase chain reaction (PCR) was performed according to standard protocol. Primers presented in Table 3 were used. Fragments were analyzed by sequencing with a sequencer ABI PRISM 3130 DNA (ThermoFisher Scientific, Waltham, MA, USA). The variants are named according to the reference transcript of the genes identified in the NCBI.

### 4.7. RNA Analysis

Total RNA was extracted from lymphocyte fraction isolated from peripheral blood using ExtractRNA reagent (Evrogen, Moscow, Russia). Total RNA was treated with DNase I (Thermo Fisher Scientific, Waltham, MA, USA), and reverse transcribed using M-MLV Reverse Transcription System (Dialat, Moscow, Russia), according to the manufacturer’s instructions.

The PCR product for deep NGS sequencing was obtained from the cDNA of the healthy control and all members of the family, including the proband, mother, and father using primers HPS1_ex2F (5′-CAGCCCTTTCTGAACCTCTG-3′) and HPS1_ex20R (5′-GCACTCTGCCCTATCCTCAG-3′). NGS libraries were prepared using the “SG GM” Kit (Raissol, Moscow, Russia) and sequenced on the FASTASeq platform in paired-end mode (2 × 150 b.p.). The targeted locus had a coverage of over 100,000×. The raw sequencing data were processed with a custom pipeline based on open-source bioinformatics tools. In brief, the pipeline involved quality control of raw reads using the FastQC tool v0.12.1, followed by read mapping to the hg38 human genome assembly and sorting using STAR 2.7.11b. Splice junctions were visualized using the Sashimi plot in IGV.

## 5. Conclusions

Hermansky–Pudlak syndrome (HPS) is an autosomal recessive disease characterized by extensive clinical and genetic polymorphism. Due to the wide variability of symptoms, diagnosing HPS presents a challenge for physicians across specialties. Key symptoms include ocular pathology (such as nystagmus, macular hypoplasia, absence of macular reflex, and hypopigmentation of the fundus), ocular or oculocutaneous albinism, and hematological abnormalities (presence of bruising, ecchymoses, gastrointestinal bleeding or prolonged bleeding during tooth replacement or surgical interventions).

In addition to these primary symptoms, our patients also exhibited unusual clinical manifestations of HPS including microphthalmia, focal pigmentation retention, and preservation of macular differentiation. The full spectrum of clinical symptoms remains unexplored due to the rarity of the disease.

The disease can present with varying degrees of symptoms and manifestations not only among unrelated individuals but also in siblings. Our cohort showed differences in the type and severity of nystagmus, iris transillumination, and bleeding, with no clear correlation to the type of mutation or affected gene.

Diagnosing HPS is often challenging due to the variability of symptom onset. Accurate diagnosis requires a comprehensive approach, including general examination, ophthalmological assessment, and detailed coagulation studies. Molecular genetic diagnosis should be based on WES to account for high heterogeneity within the syndrome and albinism. Further prognosis and patient management should involve multidisciplinary care, including evaluations by pulmonologists, hematologists, ophthalmologists, geneticists, and gastroenterologists, to ensure timely and appropriate treatment.

## Figures and Tables

**Table 1:** Comparative characteristics of clinical manifestations in different patients.

Patient	1	2.1	2.2	3	4	5	6.1	6.2	7	8.1	8.2
Age/Sex	18 y. o.	15 y. o.	9 y. o.	7 y. o.	9 months	6 y. o.	1 y. o.	4 y. o.	13 months	4 months	6 months
	Female	Male	Female	Male	Female	Female	Female	Male	Female	Female	Female
Subtype	HPS 1	HPS 1	HPS 1	HPS 1	HPS 1	HPS 1	HPS 6	HPS 6	HPS 6	HPS 9	HPS 9
Obstetric history	Normal full term	Swelling in the third trimester, delivery in the 37th week, cord contusion	Delivery in the 37th week	Delivery at 42 weeks in the head presentation	Normal full term	Normal full term	Normal full term	Normal full term; small for date	Normal full term	Normal full term	Normal full term
Nystagmus	Horizontal	Mean amplitude, horizontal	Mean amplitude, horizontal	Jerky nystagmus	Small amplitude, horizontal	Horizontal	Rotatory	Small amplitude, horizontal	Small amplitude, horizontal	Small amplitude, horizontal	Small amplitude, horizontal
Iris transillumination	1–2 grade	1–2 grade	2–3 grade	3–4 grade	1–2 grade	1–2 grade	1–2 grade	1–2 grade	1–2 grade	2 grade	2 grade
Fovea hypoplasia	2–3 grade	2–3 grade	2–3 grade	Differentiation maintained	1–2 grade	4 grade	1–2 grade	1–2 grade	1–2 grade	1–2 grade	1 grade
Hematological changes *	Tooth extraction and epistaxis	-	Ecchymosis and epistaxis	-	Fecal blood	Epistaxis and ecchymosis	-	Ecchymosis, epistaxis	-	Ecchymosis and bleeding from minor wounds	Ecchymosis and bleeding from minor wounds
ISTH-SSC Bleeding Assessment Tool score [11]	3	0	2	0	2	2	0	2	0	2	2
Light skin and eyes *	+	+	+	+	+	+	+	+	+	+	+
Hair, eyebrows and eyelashes color	White	White	White	White	White	White	White	White	Blond	White	White
Pulmonary changes *	Decreased pO2	-	Episodes of obstructive bronchitis	-	-	-	-	-	-	-	-

* Note: (+) means presence of the clinical symptom and (-) means absence of clinical symptom.

**Table 2:** Genetic variants of patients with HPS.

Patient	Gene/Transcript	Variant	Exon/Intron	Variant Type	Status	Allele Frequency (GnomAD v2.1.1)	Patient Status
1	*HPS1*/ NM_000195.5	c.1189delC p.(Q397Sfs*2) c.938-1G>A p?	Exon 13 Intron 10	Frame shift Splicing variant	Previously described [12] Novel	0.00006753 -	Compound heterozygote
2.1	*HPS1*/ NM_000195.5	c.1189delC p.(Q397Sfs*2)	Exon 13	Frame shift	Previously described [12]	0.00006753	Homozygote
2.2	*HPS1*/ NM_000195.5	c.1189delC p.(Q397Sfs*2)	Exon 13	Frame shift	Previously described [12]	0.00006753	Homozygote
3	*HPS1*/ NM_000195.5	c.1189delC p.(Q397Sfs*2)	Exon 13	Frame shift	Previously described [12]	0.00006753	Homozygote
4	*HPS1*/ NM_000195.5	c.1189delC p.(Q397Sfs*2) c.1397+135C>T p.[(Glu466Aspfs*27);(Ser459Thrfs*1)]	Exon 13 Intron 14	Frame shift Intronic variant	Previously described [12] Novel	0.00006753 0.00003186	Compound heterozygote
5	*HPS1*/ NM_000195.5	c.397G>T p.(Glu133Ter) c.972dupC p.(M325Hfs*128)	Exon 5 Exon 11	Nonsense Frame shift	Previously described [12] Previously described [12]	- 0.0002620	Compound heterozygote
6.1	*HPS6*/ NM_024747.5	c.1133T>G p.(Leu378Arg)	Exon 1	Missense	Novel	-	Homozygote
6.2	*HPS6*/ NM_024747.5	c.1133T>G p.(Leu378Arg)	Exon 1	Missense	Novel	-	Homozygote
7	*HPS6*/ NM_024747.5	c.241G>T p.(Ala81Ser) c.1457C>T p.(Ala486Val)	Exon 1	Missense Missense	Novel Novel	- 0.00005968	Compound heterozygote
8.1	*BLOC1S6*/ NM_012388.4	c.224+1G>A p?	Intron 2	Splicing variant	Novel	-	Homozygote
8.2	*BLOC1S6*/ NM_012388.4	c.224+1G>A p?	Intron 2	Splicing variant	Novel	-	Homozygote

**Table 3:** Primer pairs that are used for Sanger sequencing.

Primer Name	Sequence, 5′-3′
HPS1_1189del_F	TTAGGGTTGGCACGTCTTCC
HPS1_1189del_R	AACTTGTCCATCCTCTGGCG
HPS1_11F	CTGGCACGGGTAGAGTCAC
HPS1_11R	ATTAGCACCCCTGCGTGGAG
HPS1_5F	ACCAGATGGTCTCCAAGGTTC
HPS1_5R	CGGCATCTTATCAAACCCGC
HPS6_1F	CCACATTGGAACTGCTGGACAT
HPS6_1R	CTCCGCCGCTGGTAGTA
BLOC1S6_2F	TGGGCTCCTTTCTACCTGCT
BLOC1S6_2R	TCCACCTGAACAATCCCCAAT

## Data Availability

The datasets used and/or analyzed during the current study are available from the corresponding author upon reasonable request.
